# Omics: The way forward to enhance abiotic stress tolerance in *Brassica napus* L

**DOI:** 10.1080/21645698.2020.1859898

**Published:** 2021-01-19

**Authors:** Ali Raza, Ali Razzaq, Sundas Saher Mehmood, Muhammad Azhar Hussain, Su Wei, Huang He, Qamar U Zaman, Zhang Xuekun, Cheng Yong, Mirza Hasanuzzaman

**Affiliations:** a Key Lab of Biology and Genetic Improvement of Oil Crops, Oil Crops Research Institute, Chinese Academy of Agricultural Sciences (CAAS), Wuhan 430062, China; b Centre of Agricultural Biochemistry and Biotechnology (CABB), University of Agriculture, Faisalabad 38040, Pakistan; c College of Agriculture, Engineering Research Center of Ecology and Agricultural Use of Wetland of Ministry of Education, Yangtze University Jingzhou, China; d Department of Agronomy, Faculty of Agriculture, Sher-e-Bangla Agricultural University, Dhaka-1207, Bangladesh

**Keywords:** CRISPR/Cas9, climate-resilient rapeseed, drought, metabolomics, marker-assisted selection, systems biology, salinity, transcriptomics

## Abstract

Plant abiotic stresses negative affects growth and development, causing a massive reduction in global agricultural production. Rapeseed (*Brassica napus* L.) is a major oilseed crop because of its economic value and oilseed production. However, its productivity has been reduced by many environmental adversities. Therefore, it is a prime need to grow rapeseed cultivars, which can withstand numerous abiotic stresses. To understand the various molecular and cellular mechanisms underlying the abiotic stress tolerance and improvement in rapeseed, omics approaches have been extensively employed in recent years. This review summarized the recent advancement in genomics, transcriptomics, proteomics, metabolomics, and their imploration in abiotic stress regulation in rapeseed. Some persisting bottlenecks have been highlighted, demanding proper attention to fully explore the omics tools. Further, the potential prospects of the CRISPR/Cas9 system for genome editing to assist molecular breeding in developing abiotic stress-tolerant rapeseed genotypes have also been explained. In short, the combination of integrated omics, genome editing, and speed breeding can alter rapeseed production worldwide.

## 1. Introduction

As agriculture greatly depends on the prevailing atmosphere, the crop plants often suffer from various environmental or abiotic stress due to climate change. There are many abiotic stresses, viz. salinity, drought, cold, waterlogging, and temperature fluctuations, which considerably hinder the growth rate and reduce agricultural yield globally.^1^^,2^ As reported by USDA-FAO, drought and salt stress are important limiting components affecting about 26% and 20% of the agricultural land, respectively (American Geophysical Union; https://sites.agu.org). Soil salinity is the second major limiting factor of agriculture. Salt stress mostly affects the agricultural land of arid and semi-arid regions worldwide.^3^ Plants under dehydration-inducing conditions ultimately hinder the growth rate, decrease crop production, and encourage adverse effects on plant physiological, metabolic, and biochemical processes.^2–4^ Moreover, the effect of several other abiotic stresses such as waterlogging,^5,6^ extreme temperature,^7^ and metals/metalloids toxicity on crop productivity have also been reported in different crops, including rapeseed.^8^

**Figure 1.**
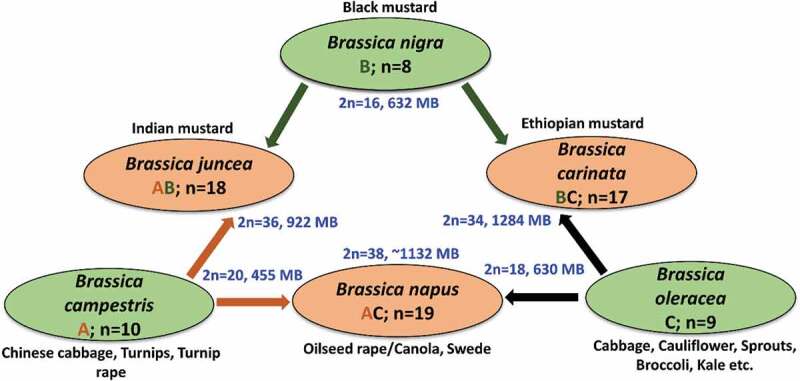
Systematic triangle of cross-breeding among economically important six *Brassica* species comprising three diploids (green) and three allotetraploids (orange) along with their estimated genome size. Letters (ABC) within the ovals denote the genomic symbols. N means the number of chromosomes. U’s triangle was created according to Nagaharu (1935).^9^ Genome size was retrieved from refs.^10–14^

**Figure 2.**
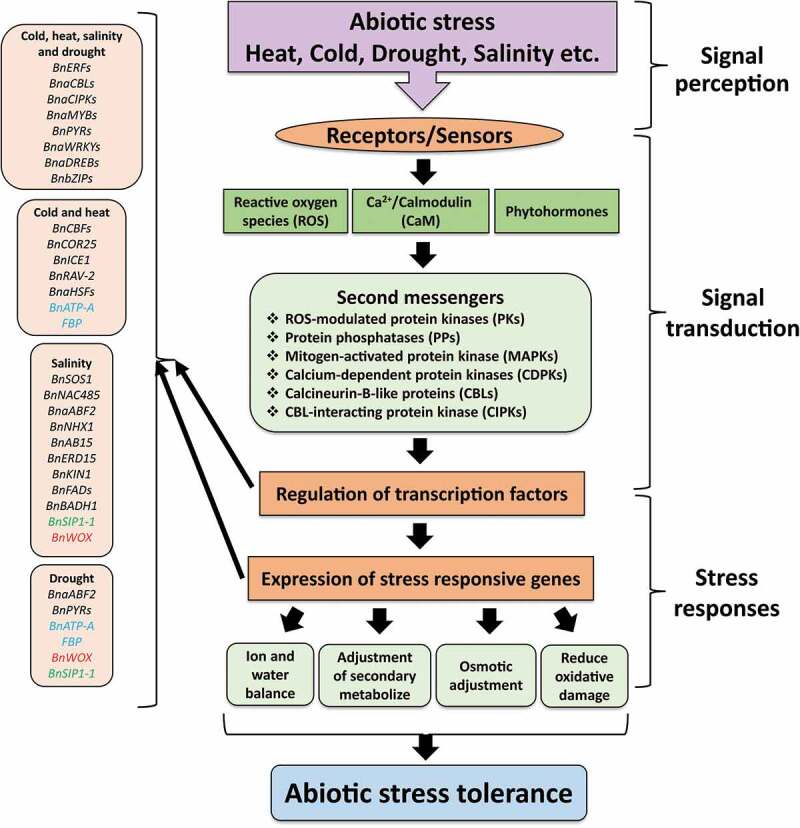
General abiotic stress signaling pathways in plants, starting from signal perception and leads toward the stress responses. Early prevalence of stress sensing via receptors/sensors cascades the downstream stress response by ROS, CaM, and phytohormones. Signal extension and transduction by secondary messengers such as PKs, PPs, MAPKs, CDPKs, CBLs, and CIPKs signaling cause differential regulation of transcription factors (TFs) and stress-responsive genes. The regulation of such TFs and genes leads to the adjustment of physiological, biochemical, and molecular responses, thus, improving stress tolerance. Boxes on the left side, indicating the essential genes and TFs that act and protect plants under extreme environmental stresses. Blue, red and green text color in the gene-boxes showing that these genes or TFs works in different stresses. Adapted and slightly modified from Raza (2020a),^8^ with permission from Springer Nature

*Brassica napus* L., also called rapeseed or canola, has emerged as an important crop globally through rigorous breeding programs during the past few decades. Among oilseed crops, rapeseed is second in world oilseed production only to soybean. It belongs to the Brassicaceae family, that have 419 genera and 4130 species worldwide. Rapeseed genome is 2 n = 38 (AACC), which arises from the genome hybridization of *B. oleracea* and *B. rapa* (Fig. 1; See refs. 14, 15) Rapeseed is now primarily grown in China, Canada, Europe as a cause of edible oil, livestock ration, and industrial derivatives.^16,17^ Rapeseed oil content is about 30.6–48.3% of the dry weight. The oil profile of rapeseed includes vital fatty acids such as oleic (56.80–64.92%), palmitic (4.18–5.01%), linoleic (17.11–20.92%) acids. The quantity of α-tocopherol is about 13.22–40.01% of the total oil contents.^18^ Due to climate change, urbanization and industrialization, abiotic stresses have become the main threat to crop production. Rapeseed, along with major crops worldwide, is frequently subjected to stresses which have significant effects on the physiological, biochemical, and molecular functions of plants, which ultimately impacting crop growth and production.^8^ Improved abiotic stress tolerance of rapeseed is a fundamental approach to increase oilseed production.^19,20^ General stress signaling pathways are described in Fig. 2.

Over the past decade, several reports indicated cloning and overexpression of the genes in rapeseed which enables plants to withstand several abiotic stresses (Table 1). Earlier, the *BnNAC485* gene related to the NAC family was cloned, and results show that *BnNAC485* was significantly expressed in 21-day-old seedling and cotyledon that was prompted by abiotic stresses.^28^ In another study, the overexpression of *BnNCED3* increases abscisic acid (ABA) accumulation, nitric oxides (NO), and generation of reactive oxygen species (ROS) in transgenic rapeseed. Furthermore, transgenic lines displayed restricted seed growth, lateral root initiation, and improvement of ABA-mediated leaf senescence by modulating ABA synthesis.^43^ Cloning and expression scrutiny of *BnICE1* revealed that *BnICE1* is vigorously linked with cold stress tolerance, particularly at low temperatures.^21^ Regulation and molecular characterization of the C-repeat-binding factor (CBF) exposed that CBF was closely connected with cold stress.^44^ Recently, the identification and expression analysis of *BnBADH1* increases salinity and drought stress tolerance,^37^ and so on. Further examples of rapeseed abiotic stress-related genes are presented in Table 1.

**Table 1.** The overview of abiotic stress-responsive genes or/and transcription factors of rapeseed along with their key responses under stress environment

Genes	Key stress responses	References
*BnICE1*	Increase cold tolerance and highly expressed in different tissues, like stem, leaf, and hypocotyls	21
*BnCBFs*	Increase cold tolerance via ABA-independent pathways	22
*BnCOR25*	Improve cold tolerance via the ABA-dependent pathway	23
*BnSOS1*	High expression of *BnSOS1* cause the exclusion of harmful Na^+^ into the apoplast area from the cellular environment and ultimately increase the salinity tolerance	15, 24
*BnaCBLs* and *BnaCIPKs*	Overexpression and mutant analysis improves tolerance to salinity, cold, heat, drought, ABA signaling, and low potassium	25–27
*BnNAC485*	Transgenic plants show resistance to salinity via ABA-mediated pathway, and it also improves the early flowering in transgenic plants	28
*BnATP-A* and *FBP*	Responsible for cold and drought tolerance	15
*BnERFs* and *BnRAV-2*	Transcriptome analysis reveals that these genes involved in cold tolerance	7
*BnERF2*-like [ERF2.4]	Improve the potential of antioxidant systems which improve resistance to submergence and oxidative stresses	5
*BnaABF2*	Improve drought and salinity tolerance via the ABA-dependent pathway	29
*BnaMYBs*	Improves tolerance to cold, heat, drought, and salinity through the regulation of ROS defense genes	30, 31
*BnSIP1-1*	Overexpression improve the growth rate under salinity, osmotic, and ABA stresses; mainly involved in osmotic tolerance	19
*BnNHX1, BnAB15, BnRD29A, BnERD15*, and *BnKIN1*	Increased salinity tolerance in transgenic plants	19
*BnbZIP13, BnbZIP28*, *BnbZIP41, BnbZIP2, BnbZIP53, and BnbZIP60*	Expression analysis indicated that these genes enhance resistance to heat and salt stress	32
*BnWOX10*, *BnWOX50, BnWOX44*, and *BnWOX18*	Increase resistance to drought, and salinity by the regulation of plant hormones	33
*BnPYR1-3, BnPYL1-2, and BnPYL7-2*	Expression analysis indicated that these genes enhance resistance to drought, salinity, and elevated temperature	4
*BnaPLD*α*1, BnaPLDδ*s *and BnaPLDδ*	Increase tolerance to salinity, cold, dehydration, and ABA response	34
*BnWRI1*	Regulating the impact of heat stress on fatty acid biosynthesis	35
*BnFADs*	Increase resistance to Cd and salt stress	36
*BnBADH1*	Increase resistance to salinity and drought stress	37
*BnKCS1-1, BnKCS1-2*, and *BnCER1-2*	Upgrade cuticular wax in transgenic plants and increase drought tolerance	38
*BnaNHXs*	Differential expression of *BnaNHXs* improves salinity tolerance [nitrogen and low phosphate]	39
*BnNRT2s* and *BnNRT2.5 s*	Increase tolerance to nutrient deficiency [phosphorus and potassium], waterlogging, and drought stress	40
*BnCOL2*	Regulating the plant response to drought stress	41
*BnNF-YA3*	Regulating the plants’ response to salinity, drought, and ABA	42

The improvement of *Brassica* spp. for abiotic stress tolerance has been addressed since a few decades, and several tools have been employed, such as identifying and manipulating crops for abiotic stress tolerance, applications of several omics tools for the elucidation of genes associated with abiotic stress tolerance.^45^ However, the major discoveries in this arena are gene discovery in response to different stress, marker development, gene mapping, transcriptomic analysis, and regulation of physiological and biosynthetic mechanisms.

The advancement of omics tools such as genomics, transcriptomics, proteomics, metabolomics, and phenomics has modernized molecular biology research. These allowed meticulous examination of connections between molecular machinery to assimilate genes, proteins, and many essential regulatory components, collectively organizing systematic molecular strategies.^46^ The genome sequencing of *Brassica* crops has allowed more understanding of crop genetics and opens new avenues for crop improvement. Moreover, it has transformed molecular and functional genomics in *Brassica* cultivars.^47^ Various transgenic rapeseed cultivars have been developed that allow more tolerance than conventional varieties. Advancement in emerging approaches for genome editing (GE) like CRISPR/Cas9 has helped explore plants’ mechanism against stress responses.^48^ This review described the integrated omics approaches that could efficiently enhance rapeseed production for global requirements and sustainable agriculture production.

## 2. The Interplay of Omics Approaches to Address Abiotic Stresses

**Figure 3.**
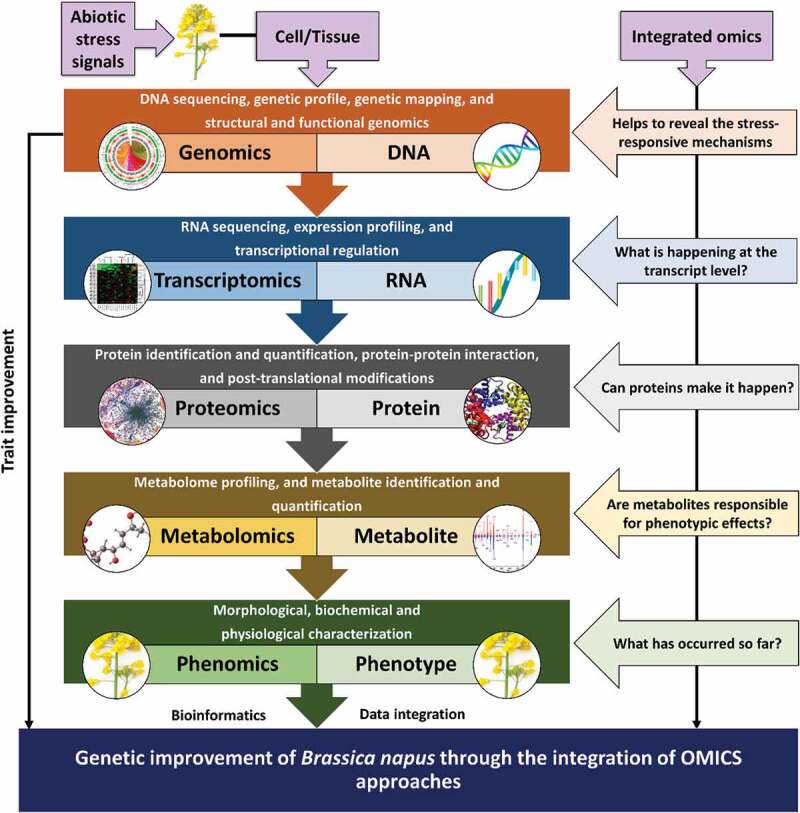
The central dogma of systems biology showing the flow of information from DNA to phenotype. Step-wise presentation of OMICS approaches for studying abiotic stress responses. Ultimately, the integration of omics tools, primarily genomics (mainly single-cell/tissue-specific) leads toward the genetic improvement of rapeseed by modulating several agronomic traits such as environmental stress tolerance, yield, plant height, seed rate, flowering, photosynthesis, and respiration rate, root and shoot length, grain quality and yield, biomass production, etc

Omics approaches have been applied to gain insight in all biological mechanisms occurring during abiotic stresses in crops (Fig. 3). Over the past few decades, omics strategies have been improved to develop stress-resistance cultivars. Hence, a paradigm shift has been brought in research by these omics strategies to tackle abiotic factors and open up new horizons for enhanced understanding of numerous features responsible for crop resistance. To combat abiotic stresses, plants adjust their omics profiles accordingly.^46^ Therefore, the association of omics techniques will help to uncover candidate genes and their biosynthesis pathways in the future. Deciphering the association among omics approaches will potentially help us to illuminate molecular pathways that are actively controlled by abiotic stresses. Additionally, these combined tools will provide a comprehensive data set for biological system analysis.

### 2.1. Genomics Approaches: Provide Insights into Stress-associated Mechanisms

Genomics tools have gained enormous importance for the elucidation of desired genes related to abiotic stress tolerance. During the last few years, significant advancement has been achieved by the researchers in the functional genomics era to scan the genomes of many crop species.^49^ Several genomics tools have been implemented in plant biology to explore the genomes of many crops for abiotic stress-tolerance, such as quantitative trait loci (QTL) mapping, which has emerged as a valuable technique to investigate the genetic diversity of crops. Moreover, up-gradation of QTL mapping and marker technology has allowed deciphering modern breeding approaches like marker-assisted selection (MAS) and single nucleotide polymorphism (SNPs).^47,50^

High-throughput sequencing techniques, such as next-generation sequencing (NGS), have made it feasible to sequence an organism’s whole genome. The integrated approach of genome-wide association studies (GWAS) and NGS technologies have provided remarkable opportunities to predict stress-related genes.^51^ Nevertheless, the whole genome comprising genic and intergenic regions can form a particular scheme for plant development. The relationship between functional, comparative, and structural genomics is incredibly desirable for organic production.^49^ New opportunities have been created by the recent improvements in crop genomics to develop abiotic stress-tolerant crops. Moreover, the availability of genomics platforms for gene identification and trait examination in rapeseed, which have enormous capabilities for molecular breeding integrated with modern tools of breeding related to GE techniques. The accessibility of a reference genome to rapeseed permits the application of such technologies in rapeseed could be predicted to attain significant progress in the future. In the subsequent sections, we have discussed the recent progress in major genomics tools.

#### 2.1.1. Marker-assisted Breeding

For breeding and crop improvement, powerful approaches like marker‐assisted breeding (MAB) and MAS have been comprehensively used to map numerous desired genes in rapeseed.^47,50,52^ Molecular markers linked with stress-responsive genes or QTLs can be recognized, which can be employed as a secondary choice principle to advance breeding proficiency via MAS. However, the practice of MAS for operating modest traits has been modernized in numerous rapeseed breeding programs. Thus, MAS for enlightening multifaceted traits appears to be at initial stages.^47,50,53^ Furthermore, to exploit the genetic diversity in different crop plants, a high throughput strategy of genomic tools is used. Rapeseed, which has a comparatively low level of genetic diversity, several studies based on genome-wide MAS have been conducted to describe the genetic variability in rapeseed.^47,50,52^

Nucleotide sequences of genes have been established using DNA markers, which offer fundamental knowledge to understand the genetic divergence of a species from primitive families and leading landraces that hold their genetic materials well preserved in gene databank for future applications.^47,52,54^ DNA markers are applied to probe the genome to screen desired loci and transgenes by repeated selective events. Certain important traits were identified, where DNA markers have been applied for genetic mapping in rapeseed.^52^ Numerous SNPs have been discovered in rapeseed using QTL mapping for desired traits.^55^ Likewise, RAPD, AFLP, ISSR, and SSR have been used to find the genetic variability in rapeseed.^56–59^ Detailed information will be presented in the subsequent sections (2.1.1.1-2.1.1.7) to highlight the efforts in rapeseed mapping using different approaches.

##### 2.1.1.1 The contribution of RAPD markers

PCR-based random amplified polymorphic DNA (RAPD) markers primarily comprised on random amplification of segments of polymorphic DNA.^60^ Previously, several studies have been carried out using RAPD markers for rapeseed mapping for different traits. For example, a double haploid (DH) population resulting from the F_1_ generation of the cross Apollo (black-seeded) x YN90-1016 (yellow-seeded) rapeseed was examined to detect molecular markers related to the yellow-seed trait.^61^ An essential gene (*pigment 1*) lined by 8 RAPD markers was linked with the yellow seed coat color trait in the F_1_ generation. This gene described 72% phenotypic difference in seed coat color.^61^ Likewise,^62^ examined the F_2_, BC_1,_ and F_1_-derived DH population for seed color using 810 RAPD and 512 AFLP markers. Out of these, 240 RAPD markers showed polymorphisms among the parents. Additionally, 4 RAPDs and 16 AFLP primer pairs displayed polymorphisms among the bulks, whereas only 2 RAPD and 8 AFLP markers were detected in the locality of the seed coat color.^62^

Furthermore, a polymorphic marker linkage map conveying nine linkage groups (LGs) was created from F_2:3_ populations of rapeseed [SLMO46 (winter type and cold-resistant) x Quantum (spring type and cold susceptible)] using 47 RAPD markers.^63^ They identified one presumed QTL for winter survival elucidating only a 5% phenotypic difference. The QTL was located on LG6 is amid 670 and 650 bp sized RAPD markers with 4 cM distance from the 650 bp marker and had adverse conserving consequence.^63^ In another study, a similar rapeseed population by 47 RAPD and 32 SSR markers, a linkage map conveying 14 LGs was prepared and presumed QTLs were identified on 3, 8, 9, and 10 LGs. These QTLs explained a 24% phenotypic difference in the freezing tolerance in the studied rapeseed population.^64^ Besides, RAPD markers have been extensively used to evaluate genetic diversity and polymorphism between different rapeseed genotypes/lines/populations.^58–59–65–68^

##### 2.1.1.2 The contribution of AFLP markers

Amplified fragment length polymorphism (AFLP) is a PCR-based technique in which a subgroup of constraint remains are selectively amplified with oligonucleotide primers opposite to sequences. It can also be applied to fresh and partly decade-old DNA.^69^ Previously, the separation of the rapeseed *Bnms3* gene (responsible for recessive genic male sterility) for evolving precise PCR markers for MAS and understanding this gene’s crucial function has been investigated. Notably, the *Bnms3* gene was labeled with seven AFLP markers; out of these, six AFLPs were recognized to be co-segregated with the target gene in a population of 115 individuals.^70^ In another study, *Brassica* 60 K Infinium SNP array, AFLP, and SSR markers were used to make a high-density genetic map in the DH rapeseed population (Zhang et al. 2014c). Using the genetic map, a novel QTL (*qBEC-A3a)* for boron deficiency was identified, and a similar map was then employed to identify different QTLs related to plant growth (36 QTLs with 6.14–46.27% phenotypic difference) and boron uptake (12 QTLs with >10% phenotypic difference). These findings provided vital QTLs suited for fine mapping and the actual markers for improving breeding productivity in rapeseed.^71^

Moreover, Fan et al. (2015)^72^ described the QTLs’ mapping related to waterlogging and drought resistance in the DH rapeseed population’s seedling stage via AFLP and SSR markers. Significant genetic diversity was detected in resynthesized rapeseed lines using AFLP markers compared to the gene pools of conventional spring and winter rapeseed genotypes.^73^ In the next experiment, these authors correlated the genetic diversity with the heterotic yield in trial hybrids.^74^ Later, the yield characters of 184 F_2:3_ lines were accessed via AFLP and SSR markers. Results show that 73 markers were linked with 12 yield characters with an involvement rate of 3.54–15.88% and an average of 6.60%. The genetic distance was expressively associated to heterosis of F_1_ trials via 66 markers related to yield character and suggesting that the correctness of yield and heterosis estimate reliant on QTLs linked with yield characters in rapeseed required to be upgraded.^75,76^ On the other hand, AFLP markers have been extensively used to access the genetic diversity among different rapeseed genotypes/lines/populations.^56,58,77^

##### 2.1.1.3 The contribution of SSR markers

Simple sequence repeats (SSRs) or microsatellites are short tandem repeated motifs of 1–6 nucleotides present in huge amounts and may fluctuate in the number of repetitions at a specified locus. These markers have numerous benefits over other markers, such as hereditary co-dominance.^78^ The sum of freely accessible *Brassica* SSR primers is growing because of openly sponsored international enterprises (www.brassica.info). Nevertheless, compared to other imperative crop plant species, limited SSR markers are easily accessible, meaning few consensus maps have been described in DH rapeseed populations.^79^ The publicly available set of vigorous, extremely polymorphic, mapped SSR markers covering the entire rapeseed genome have helped rapeseed genetics researchers in mapping and genome amalgamation.^80^

During the past few years, significant progress has been made using SSR markers in integrating available and new genetic maps. Further, the QTL alignment, playing a crucial role in correlation of candidate genes with QTLs. For instance, using F_2:3_ rapeseed populations,^64^several major QTLs were detected on 3, 8, 9, and 10 LGs with a phenotypic difference of 24% in freezing tolerance. SSR markers, together with *Brassica* 60 K Infinium SNP array, were used to make a high-density genetic map in the DH rapeseed population under boron deficiency. This resulted in 12 QTLs with >10% phenotypic difference for boron uptake and a novel QTL linked with the growth and boron deficiency (Zhang et al. 2014c). In rapeseed, 53 SSR markers were expressively linked with 3 phenolic sections, and 11 markers were connected with total phenolic acid contents. Out of these, only 4 SSR markers result from QTL for seed color by association mapping.^81^ Additionally, 25 and 11 SSR markers are related to seed coat color and oil content, respectively. In contrast, only six SSR markers are connected to both oil content and coat color.^82^ In a recent study, 36 SSR markers have been used to examine the variability between 41 rapeseed lines linked with drought tolerance QTLs.^83^ Apart from the above-mentioned traits, SSR markers have shown great potential in deciphering genetic diversity between rapeseed genotypes/lines/populations [58, 84, 85, 86].

##### 2.1.1.4 The contribution of ISSR markers

The commonly used PCR-based markers such as RAPDs, AFLPs, and SSRs have some limitations like low reproducibility, high cost, and need flanking sequences. Therefore, inter simple sequence repeat (ISSR) is an updated method that incapacitates these limitations. This method combines numerous advantages of AFLP- and SSR-based investigations with the universality of RAPD.^86–88^ Water-stressed rapeseed cultivars showed maximum discrepancy subsidized by ISSR markers went to relative water content (78%) at normal state, to root/shoot index (66%) at modest stress state and to root length (53%) at severe stress environment.^89^ Recently, while studying drought tolerance signals and their relationship with ISSR markers in rapeseed genotypes, most of the ISSR markers were found to be linked with some of the drought tolerance indices.^90^ ISSRs have been effectively employed in the genetic diversity studies at inter and intra definite levels in numerous plant species, including rapeseed.^89,91,92^ Molecular mapping using ISSRs linked with QTLs or candidate genes is yet to be reported in rapeseed, while this has been achieved in some other crop plants.

##### 2.1.1.5 The contribution of SCAR markers

To lessen the difficulties that rise when consuming conventional markers such as RAPD, ISSR, IRAP, AFLP, SSR, etc., the sequence characterized amplified region (SCAR) marker was established. These markers can be derived from ISSR, IRAP, and RAPD markers. It signifies a particular, distinct genomic DNA part that was noticed in PCR extension with a pair of definite primers.^93–95^ These markers have great potential in plant identification at inter- and intra-specific species or population level.^93,96,97^ Previously, the linkage mapping using SCAR markers has been linked with 18-carbon fatty acids in rapeseed. Developed SCAR marker (L1L9) shows about 25% genetic difference for 18-carbon fatty acids in rapeseed.^98^ Later, using AFLP, SCAR markers have been developed for the trait-specific gene. For instance, SCAR markers have been characterized to be linked with a dominant genic male sterility [suppressor gene (*Rf*)] in rapeseed by several researchers.^99–101^ Additionally, SCAR markers have been associated with self-incompatibility in rapeseed and showed significant results at a polymorphic level compared to CAPS markers.^102^ The above-mentioned markers were handy for improving different traits and accelerating MAS progression in rapeseed breeding plans. For cultivar fingerprinting, transposon insertion-SCAR markers have been characterized in rapeseed. These markers can be considered a vital tool in the reverse genetic scheme for separating novel genes in rapeseed.^103^

##### 2.1.1.6 The contribution of CAPS markers

Cleaved amplified polymorphic sequences (CAPS) markers are PCR-amplifications of DNA remains with specific primers after the digestion of restriction enzymes and the harvests’ isolation in an agarose gel. Practical CAPS markers can be established on a target gene’s identified sequence to analyze its regulation, structure, purpose, and expression. These markers are closely connected with the target gene and are particularly supportive of MAS.^104,105^ Earlier, SCAR and CAPS markers were developed and mapped to be associated with seed coat color gene in a resynthesized purely yellow rapeseed line obtained from the F_1_ DH population using a genome-walking method.^106^ In a recent study, the SCAR and CAPS markers were linked with *Leptosphaeria maculans* resistance gene *Rlm6* in *B. napus* x *B. juncea* interspecific hybrids. Segregation of both markers related to the *Rlm6* gene was established by using F_2_ and F_3_ progeny. Interestingly, the segregation of CAPS markers and phenotype for blackleg disease cruelty in the F_2_ population had a Mendelian ratio of 3:1 in resilient vs vulnerable plants, respectively, suggesting that the genetic regulator of resistance was carried by a dominant gene.^107^

In a MAS analysis of new high oleic and low linolenic winter rapeseed inbred lines, genotyping was completed for the assortment of homozygous lines via allele-specific CAPS markers and SNaPshot assay. Lastly, new high oleic and low linolenic winter rapeseed recombinant lines were obtained, ready to be used as a preliminary material in developing new varieties with high oil value than traditional types.^108^ In another study, CAPS markers were used for the identification of two mutant alleles (*BnaA.FAD2* gene) in rapeseed. Further, CAPS markers also provided relatively good results while detecting the heterozygosity in *BnaA.FAD2* gene.^109^ These examples indicate the importance of CAPS markers in MAS studies to identify and develop new breeding lines.

##### 2.1.1.7 The contribution of SNPs

Genomes of all the organisms consist of single base pairs sequences called SNPs. Rapidly decreasing DNA charges for re-sequencing and accessibility of a more advanced SNP platform for rapeseed open up new horizons for significantly more effective gene mining and genetic mapping.^47^ More than 75% of detected SNPs were useful and could be exploited in modern molecular breeding schemes.^47,110^ Likewise, SNP analysis was documented to study the heat stress on different rapeseed varieties under field conditions, and data were recorded from flowering to maturity phase for different agronomic traits. Association mapping (AM) for various agronomic traits was executed to detect heat-related QTLs. For these 37,269 SNP markers were employed and showed that several genes were strongly correlated with abiotic stress-tolerance in rapeseed.^111^ Numerous examples are presented in Table 2 and the next section.

**Table 2.** Events performed for the detection of SNPs in rapeseed

No. of genotype used	Sequencing method	SNP calling tool	No. of SNPs	References
2	Solexa sequencing Illumina	MAQ	23,330–41,593	112
8	Illumina GAIIx system	RADtags	20,835	113
84	Illumina mRNA-Seq	Trinity	101,644	114
4	Illumina GoldenGate assay and Illumina Infinium	MIRA	5764	115
4	Illumina Infinium™ 6 K	SGSAutoSNP	5306	116
52	Illumina HiSeq 2000 platform	FaSD, Freebayes and SAMtools	4.3 Million	117
437	Brassica 60 K Illumina Infinium array	CLC Genomics Server	52,157	118

#### 2.1.2. Evidence of QTL and GWAS Studies

A GWAS is a method employed in genetics investigation to associate definite genetic differences with particular traits in different individuals. It overwhelms numerous restrictions of traditional gene mapping (QTL) by providing advanced resolution, often to the gene level, and consuming trials from formerly investigated populations in which frequently happening genetic differences can be connected with a phenotypic difference. The start of high-density SNP typing permitted whole-genome examinations to recognize often insignificant haplotype hunks that are pointedly associated with quantitative traits.^119,120^ For instance, Jian et al.^121^ studied the recombinant inbred lines (RIL) population at the seedling stage under salt and drought stress for GWAS. Notably, 2795 SNPs were detected, and the analysis revealed that numerous minor-effect loci regulate the germination percentage of rapeseed, and the various gene is activated under drought or salinity stress. Furthermore, a GWAS experiment was performed to exploit the QTLs correlated with salinity resistance in rapeseed. Genotyping was carried out through *Brassica* 60 K Illumina Infinium SNP arrays and detected 75 SNPs found on 14 chromosomes. Moreover, 38 candidate genes were evaluated, triggered under salt stress and belongs to different groups such as enzymes, transporters, aquaporins, and transcription factors (TFs). This experiment permits examining the molecular pathway of salt-resistance and may help MAB for salt tolerance in rapeseed.^51^ Recently, AM was done at the seed germination stage for 214 rapeseed inbred lines to locate QTLs linked with salinity tolerance. The genotyping was performed using 60 K *Brassica* Infinium® SNP platform, and 110 SNPs were detected with salt stress regulation. Furthermore, 56 targeted genes were mapped in the close locality of QTL regions. Thus, this result provides substantial proof for evaluating rapeseed salt tolerance’s genetic regulation and may support MAB for salt resistance in rapeseed.^122^

Genetic mapping of QTLs is the 1^st^ phase for discovering the genetic design of a trait and in executing MAS in plant breeding scheme.^123^ Stress tolerance results from multiple responses/mechanisms, assessment of these mechanisms and stress-related QTLs has assisted plant breeders in developing elite genotypes with stress-tolerance. Genetic investigations for the development of abiotic stress-tolerance rapeseed have been extensively studied for many stresses, as shown in Table 3.

**Table 3.** Experiments performed for the identification of QTLs in rapeseed under stressful environment

Stress type	No. of lines used	Genotyping/Linkage map	No. of QTLs	Chromosomal location	Key findings	References
Freezing	199 F_2:3_ lines	350 SSR and 250 RAPD	4	8, 6, 15, and 14 based on BBSRC maps	Identified QTLs shows 24% of phenotypic variances and with the help of NGS, these QTLs could use in MAS freezing tolerance	64
Waterlogging and drought	150 DH-lines	183 SSR and 157 AFLP	28-CK, 26, and 31 for stresses	A1	A few QTLs for both stresses overlapped each other, suggesting that genetic bases for DH were similar to some extent in rapeseed	124
Salinity	85 inbred-lines	Illumina Hiseq-2000	62	A1, A2, A3, A5, A7, C3, and C9	Identified QTLs strongly associated with ion-homeostasis, shoot biomass, and salt-tolerance via AM, and *BnaTSN1* as a candidate gene for salt-tolerance	125
Drought	225 DH-lines	*Brassica*-60 K Illumina Infinium array	20	A01, A06, A08, C01, and C03	Identified QTLs colocalized to 2 main QTLs, and showing that compensation is genetically controlled due to a powerful correlation between the morphology of root, productivity, and time of flowering	126
Drought	IMC106RR and Wichita	Illumina HiSeq-2000	1	A10	Candidate gene *Bna.FLC.A10* were identified through polymorphism, and the results give a sight of genome-wide variation among rapeseed with enhancing information about the root genetic basis under drought stress	127
Drought, salinity and low-temperature	ZS11	Microarray and EST-based analysis	31	A02, A03, A06, A08, A09, C01- C07, and C09	26 *Bna*WRKY genes were triggered by multiple stresses demonstrating that WRKY genes have a pivotal role in regulating multiple stresses	128
Salinity	196 F_2:3_ lines	532 molecular markers	45	A1–A10 and C1–C9	*Bna003640* was found to be the trigger in salinity and regulate the salt-tolerance mechanism	129
Salinity	368	*Brassica* 60 K Illumina Infinium SNP array	25	A3, A9, C5, C6, and C7	38 candidate genes were evaluated, which triggered under salinity and examined the molecular pathway which may help MAB for salt-tolerance	51
Salinity	196 F_2:3_ lines	IP and SCAR markers	1	A10	Lobed leaf gene (*Bra009510*) gene was mapped, and results presented that this gene might regulate the salt stress -tolerance	130
Cold	147 F_2:3_ lines	333 SSR markers	11	A08	Identified QTLs showed the phenotypic variation of 42.50% and 1.09%, and two genes *BnaA08g15470D* and *BnaA08g05330D* have been strongly linked with cold-tolerance	123

### 2.2. Transcriptomic Approaches: What Appears to Be Happening at TheTranscript Level?

Transcriptomic is a very promising technique to understand abiotic stress regulation in plants to decipher novel genes and various regulatory networks through transcriptome profiling.^131^ Transcriptomic or/and gene expression investigation by next-generation sequencing (NGS), RNA-seq profiling, subtractive libraries, expressed sequence tags (ESTs), serial analysis of gene expression (SAGE), and microarray have great potential to improve genomic resources of plants such as gene discovery and functional analysis.^132,133^ Recently, numerous rapeseed genome sequencing and re-sequencing projects have been completed (Supplementary Table 1). Based on the NGS technologies, a ***Brassica napus* pan-genome information resource (BnPIR)** database http://cbi.hzau.edu.cn/bnapus/index.php) has been developed to facilitate the *B. napus* researchers in the post-genomic era. Global transcriptome profiling provides an excellent opportunity for comprehensive knowledge about gene expression and could help identify numerous regulatory mechanisms. In the subsequent segments, we have explained the potential of widely used approaches for studying gene expression at the transcript level. Table 4 shows a summary of transcriptome-based experiments performed in stressful conditions.

**Table 4.** Summary of some transcriptomic studies conducted under stressful environment in rapeseed

Approach	Stress	Objective	Genotype	Tissue condition or stages	No. of DEGs and TFs, respectively	Key findings	References
RNA-seq	Waterlogging	To identify the mechanism of waterlogging tolerance	ZS9	Roots at 0 h and 12 h of stress	4432	Protein degradation is associated with the negative regulation of waterlogging	134
RNA-seq	Salinity	Regulation in leaves and roots in response to salinity	N119	Leaves and roots 1 h and 2 h after stress	14,719, and 582	Identified genes influenced by salinity in roots and leaves, a novel TFs *S1Fa*-like reported	135
RNA-seq	Drought	To decipher the candidate genes for drought tolerance	ZY821	Six-leaf stage	3657	DEGs were triggered under drought stress and providing new genes	136
RNA-seq	Salinity	To elucidate the relationship between salinity and Strigolactones	ZS11	Root and shoot after 7-days of treatment	2162-roots, 5935-shoots DEGs	Strigolactones improves salt-tolerance and results provided stress associated novel genes	137
RNA-seq	Drought, cold, salinity, heat and ABA	To decipher the function of TF families under stresses	ZS11	7-days old seedlings at 12 h after treatments	315, and 2167	About 80% DEGs of the identified 5 TFs families *Bn*AP2/ERF, *Bn*bZIP, *Bn*MYB, *Bn*NAC, and *Bn*WRKY triggers abiotic stresses	133
RNA-seq	Low temperature	To identify candidate genes responsible for fast germination	Ganyouza No. 5 and Huawanyou No. 4	1, 2, and 3-days after treatment	9111-down and 10,233-up-regulated DEGs	Many TFs such as ERF, NAC, DREB, B3, MYB, EFR, bZIP, and WRKY were also found to regulate the low-temperature tolerance in rapid germination cultivar	138
RNA-seq	Cold	To revealed conserved and novel cold-responsive genes	HX17 and HX58	After 14-days of treatment, third leafs	47,328	Two conserved (the primary metabolism and plant hormone signal transduction) and two novels (plant-pathogen interaction pathway and circadian rhythms pathway) pathways were significantly enriched with DEGs	139
RNA-seq	Cold	To explore the molecular mechanisms in different rapeseed ecotypes	Five winters and five spring ecotypes	Leaves were harvested at 0 and 12 h after treatment	25,460 and 28,512 DEGs in spring and winter oilseed ecotype, respectively	Lipid, ABA, secondary metabolism, signal transduction, and transcription factors may play vital roles in both ecotypes under cold stress	140
EST	Drought	To identify stress-responsive regulatory network	DH-12,075	Four-leaf stage	17 TFs	One protein phosphate, eight kinases, 26 regulatory genes, and 17 TFs were investigated for increased transcript level; new miRNAs and regulatory genes modulating drought stress	141

#### 2.2.1. Modern Transcriptomic Analysis by RNA-seq

RNA-seq is an emerging approach that exploits or/and offers a different outlook to examine the transcriptome sequence by granting complete access to transcripts. Therefore, RNA-seq can be conducted as a substitute strategy for many diverse techniques for quantification of the transcript with the advantage of higher sensitivity and ability to differentiate among related gene paralogs, which are different by just a small number of nucleotides.^133,135^ For instance, five TFs families have been characterized and described under various abiotic stresses in rapeseed. Totally 2167 TFs from 5 different families were documented in rapeseed; i.e., 518 *Bn*AP2/ERF, 252 *Bn*bZIP, 721 *Bn*MYB, 398 *Bn*NAC, and 278 *Bn*WRKY, including some unique transcripts as compared to actual results. Many vital representatives of these TFs and regulatory systems associated with various abiotic stress conditions have been discovered. The investigations in rapeseed against stress tolerance have extended our knowledge toward TFs. RNA-seq represents that more than 80% of TFs triggers abiotic stress, and 315 TFs (DEGs) have been observed, and these 315 DEGs were highly expressed.^133^

Yong and colleagues performed RNA-seq for the comparative transcriptomic study of rapeseed under increased salt stress. Roots and leaves were collected after 1 hour, and 12 hours of salt stress and *de novo* transcriptome sequencing yielded a total of 14,719 DEGs. Additionally, Kyoto Encyclopedia of Genes and Genomes (KEGG) and Gene Ontology (GO) enrichment analysis found that 438 transporter genes with 582 TFs were influenced by salinity in roots and leaves. DEGs under salinity provide full knowledge for rapeseed improvement.^135^ RNA-seq was achieved to elucidate the candidate genes and decipher the molecular pathways that govern the tolerance mechanism under drought stress in rapeseed. Illumina Hiseq 2000 was used for RNA-seq analysis and assemble 28,378,899 and 26,192,312 high-resolution reads. RNA-seq analysis identified 3,657 transcripts triggered by exposure to drought stress.^136^

Recently, RNA-seq was executed to elucidate the transcriptional modulation process for the fast growth of rapeseed. Various transcriptomic assemblies were established under cold stress and normal temperature with a slow and rapid germination speed rate. Transcriptome analysis showed 9111 and 10,233 DEGs, which were down and up-regulated under cold stress. Moreover, many TFs such as ERF, NAC, DREB, B3, MYB, EFR, bZIP, and WRKY were also found to regulate the low-temperature tolerance in rapid germination cultivars. These results will help future breeding programs to develop abiotic stress tolerance rapeseed genotypes.^138^ In another study, under cold stress, RNA-seq was used to explore the molecular mechanisms in different rapeseed ecotypes. They identified 25,460 and 28,512 DEGs in spring and winter oilseed ecotype, respectively. Identified DEGs mainly fitted to lipid, ABA, secondary metabolism, signal transduction pathways, and these signaling pathways may play dynamic roles in both ecotypes under cold stress.^140^ See Table 4 for more examples.

#### 2.2.2. Expressed Sequence Tags (Ests)

ESTs signify a foundation for discovering unique gene structures that provide an outline for further significant studies, like expression system, gene maps, and cDNA sequencing projects. ESTs have been considered the precise technique to unveil sequences-related knowledge under abiotic stress conditions.^142,143^ The ocsESTdb database provides information about seed ESTs and full-length CDS sequences of oilcrops seeds. The relative investigations of the oilseed databank showed that ESTs are accessible at the database of OCRI-CAAS, Wuhan, China (http://www.ocri-genomics.org/ocsESTdb/).^144^ Similarly, overtly available *Brassica* ESTs contigs equivalent to a variety of gene functions (http://brassica.nbi.ac.uk/array_info.html).

Nevertheless, 343 WRKY domains with 287 genes were documented in response to several abiotic stresses in rapeseed. Microarray and EST-based analysis showed that 74 WRKY genes had been expressed beneath stress conditions. After QTL mapping, 77 WRKY genes have been reported in 31 QTL loci associated with many stress resistance. Twenty-six *Bna*WRKY genes which are triggered by drought, salinity and low-temperature stress.^128^ Recently, available data of ESTs were examined and found 25 creatine phosphokinase (CPK) genes. A DH line of rapeseed was used to clone the cDNA sequences of 23 genes. Phylogenetic analysis was performed, and green fluorescence protein was used as a reporter gene to detect five candidates in *Bna*CPKs. Additionally, the expression of 21 *Bna*CPKs was investigated for oxidative, low potassium, abscisic acid, heat, cold, drought, and salinity stress regulation via qRT-PCR. The results suggested that multiple signaling pathways govern CPKs in response to several stresses in rapeseed.^26^ Rapeseed EST profiling was carried out to find the function of typical AP2/ERF TFs under biotic and several abiotic stress regulations.^145^ Table 1 documents the other TFs in response to abiotic stresses in rapeseed.

#### 2.2.3. Serial Analysis of Gene Expression (SAGE)

SAGE has been extensively used to investigate gene expression. SAGE method calculates a tag, which shows a product of gene transcriptome. SAGE is considered the most economical and high-throughput approach, including mRNA isolation, cloning, and sequencing. In the SAGE technique, `tag` is a short length nucleotide sequence with a pointed head-to-head specific restriction enzyme. Therefore, SAGE represents gene expression in digital form. There are many SAGE applications in plants, such as the interaction between host and pathogens, plants’ responses in several abiotic stresses, transcriptome profiling, and metabolism of many hazardous compounds.^146,147^ About functional genomics investigations of oilseed plants with the importance of seed production and metabolism mechanism of fatty acids, a database was established named Shanghai RAPESEED database (RAPESEED, http://rapeseed.plantsignal.cn/). The rapeseed has 8462 ESTs, including 2526 full-length cDNA; 8095 expressed genes, and 23,895 SAGE tags during seed growth. In RAPESEED database, all the relevant data is stored in the form of nucleotides and protein sequences.^148^ This technique has been used in several crops but needs to be considered for rapeseed in future work.

#### 2.2.4. Microarray

cDNA microarrays are used for observing gene expression and offer a proficient strategy to evaluate the potential functions of numerous genes.^149^ Microarray cDNA investigations of gene expression in various plants beneath several abiotic factors have been widely documented.^150,151^ Gene databank accessibility gave plant gene expression from microarray investigations like Gene Expression Omnibus (https://www.ncbi.nlm.nih.gov/geo/) and ArrayExpress-functional genomics data (http://www.ebi.ac.uk/arrayexpress).^152^

Microarray analysis was demonstrated in rapeseed to decipher the function of CBF/DREB1-like TFs under cold stress. Microarray and northern blot analysis confirm that the accumulation of cold-responsive genes that have a function in photosynthesis. Additionally, some other genes, such as *GLK1* and *GLK2*-like TFs, responsible for chloroplast development, trips-p/pi translocator, and β-amylase, were also produced. Hence, increased exchange rate of malate/oxaloacetate, enhancement in glycolysis, more sucrose and starch production, improved Calvin cycle enzymes, and rectification of photosynthetic efficacy was also detected, indicating that *BnCBF* hyper-expression has moderately imitated freezing-induced tolerance. Resulting in the overexpression of *DREB1/BNCBF* has shown improved not only cold tolerance but also controlled chloroplast development in rapeseed.^153^ Therefore, it is essential from an economic point of view to increase rapeseed varieties with freezing tolerance (3–5°C) using CBF genes by means of the addition of millions of acres of lands to rapeseed-production areas worldwide.^154^ A comprehensive analysis of genes triggered by drought and salinity was performed by microarray to investigate the genes that govern abiotic stress reaction in rapeseed. The results indicated that out of 536 clones, 189 and 141 clones were responsible for gene supersession under salt and drought stress, while 288 and 172 were identified for drought and salinity resistance genes, respectively. The functional examination revealed that these genes play a vital part in the growth, abiotic stress-tolerance, hormone response, signal transduction, regulatory factors, and various metabolic activities in rapeseed.^155^

### 2.3. Proteomics Approaches: What Makes It Happen?

Protein components of any living organism at a specific time are termed as proteome of that organism. At the beginning of 2000, due to the accessibility of whole-genome sequencing and mass spectrometry (MS) approaches, the proteomics strategy has been established successfully. To elaborate on the relationship between plant proteome with surrounding environmental stimuli, relative expression at the proteomic level can be studied by subjecting the plants to normal and abiotic stress conditions.^156,157^ During the last decade, yeast two-hybrid (Y2H), matrix-assisted laser desorption ionization (MALDI) and electrospray ionization (ESI)-MS, and one/two-dimensional gel electrophoresis (2-DE) have gained much attention as excellent tools for separation and as analytical approaches in proteomics analysis. In the sub-sections, we have explained the potential of these approaches.

During abiotic stresses, the protein profiling of drought and salt stress of rapeseed has been conducted to detect the cross-talk between the cells through multiple reaction monitoring. Proteins related to phosphorylation mechanisms, such as CTRI, CDPK21, and TPR, have been identified under salt stress conditions. Similarly, BSL and STN7 proteins were also recognized, which have a role in the phosphorylation process under drought stress.^20^ Nevertheless, an iTRAQ-proteomic analysis was carried out for rapeseed roots under waterlogging stress, and 7736 proteins were identified. These proteins function differently in response to stress and provide insight knowledge for adaptive mechanisms.^158^ An experiment was performed on rapeseed for quantitative proteomic profiling under salt stress self-compatibility. Self-compatibility induced by salt stress identified some unique proteins, which elucidate the molecular mechanisms underlying the breeding of rapeseed cultivars.^159^

#### 2.3.1. MALDI-TOF-MS and ESI

Nowadays, proteomics and metabolomics analysis of endogenous plant components have become very popular, and to carry out these investigations, a powerful tool such as MS imaging has been applied widely. Matrix-assisted laser desorption ionization (MALDI) and electrospray ionization (ESI) are mass spectrometry methods that have been extensively implemented for protein analysis.^160,161^ MALDI-TOF-MS and ESI are well-established approaches to analyze the cell lysate of protein contents, separated by m/c ratio.^162^ Ionization in MALDI can also be coupled with time of flight (TOF) analyzer.^164^

Recently, a study has been reported on rapeseed leaves, in which proteomic matrix, physiological and biochemical variations were evaluated under salt and lipoid acid stress. Comparative proteomic investigations were carried out for control-grown leaves as well as leaves from plants subjected to salinity combined with exogenous lipoic acid (LA) application. The proteins of various sizes were dispersed using 2-DGE and found that 28 proteins have been expressed under stress conditions. Furthermore, for the exact confirmation of these 28 proteins, MALDI-TOF/TOF MS has been used and recognized 21 proteins.^163^ 2-DGE was used for comparative proteomic analysis of thermos-sensitive genic male sterility genotypes of rapeseed during microspore and microsporocyte formation. MALDI-TOF MS has identified 28 protein, from which ten protein expressed during microsporocyte while the remaining 18 protein expressed in the microspore phase.^165^

#### 2.3.2. Two-dimensional Gel Electrophoresis (2-DE)

In modern proteomic studies, 2-DE is regarded as the driving force. Despite the dominance of MS, this strategy still holds prime importance for proteomic analysis. In various protein separation methods, 2-DE is most promising and accepted as a separation method and an analytical approach because of its powerful separation ability.^166^ For example, 2-DE was used to decipher the molecular networks regulating the salt stress in rapeseed. A salinity susceptible cultivar of rapeseed was subjected to different salt treatments and found that Na^+^ content and proline concentration were enhanced in leaf. Therefore, K/Na^+^ percentage, plant height, and shoot dry weight were reduced. 2-DE detected 110 spots on gels, and 37 of them indicated significantly abundant variations. Proteins were analyzed by employing LC-MS that has a function in photosynthesis and energy production. The results unveiled the reduction in energy production enzymes due to salt stress, whereas the increased accumulation of phosphoribulokinase under salinity in rapeseed.^167^

2-DE was run to examine the physiological and proteomic regulations of rapeseed under salinity. Seedlings were subjected to different salt stress concentrations that result in reduced photosynthesis activity and growth arrest, and 44 proteins were detected by the spots which contained many salt-resistance proteins. The results showed that the highly expressed proteins, which regulated tolerance, damage repair, and protein metabolism, might mitigate damaging impacts of salinity on respiration, energy production, photosynthesis, and chlorophyll synthesis in the leaves of rapeseed. This will help improve the understanding of the various molecular mechanisms operating under salinity in rapeseed and aid in developing GE rapeseed with increased salinity tolerance.^168^ Recently, 2-DE was used for the separation of root proteins, while MALDI-TOF-MS was employed for protein identification under salinity. Different protein spots were detected and their abundance was significantly influenced by salinity. Functional studies demonstrated the nine categories of DEPs, while 14 protein categories were detected in tolerant genotypes. The most important findings are the DEPs associated with energy metabolism, redox regulation, heat shock proteins, fructose synthesis, and glycolysis mechanism was enhanced only in the salt-tolerant genotype rapeseed.^169^ There are few databases available for proteomic analysis by using 2-DE, including rapeseed, rice, banana, tobacco, and *Arabidopsis* having protein profiles present at SWISS-2D-PAGE (https://world-2dpage.expasy.org/swiss-2dpage/) and WORLD-2D-PAGE (https://world-2dpage.expasy.org/list/).^170^

#### 2.3.3. Yeast Two-hybrid (Y2H)

Y2H system is a handy technique for genetic mapping and to exploit the various mechanism basal in protein–protein interactions by activating the reporter genes.^171^ Y2H assay was conducted to identify the various amino acids having significant potential in modulating drought stress resistance factors like DREBs in rapeseed.^172^ Y2H and bimolecular fluorescence complementation (BiFC) were executed for identification and cloning of protein phosphatase type 2 C (PP2C) and basic leucine zipper (bZIP) from rapeseed. It showed the interaction between *Bna*PP2Cs and *Bna*CPKs and unveiled the novel interaction among *Bna*CPK.^26^ Y2H system was used to study the interaction among various CBLs and CIPKs proteins under several rapeseed abiotic stresses. Results show that 23 CIPK and 7 CBL genes were detected from rapeseed database research and cloning of 17 CIPKs and 6 CBLs cDNA sequences. Green fluorescence protein (GFP) was used to determine the subcellular detection of 2 *Bna*CIPKs and 3 *Bna*CBLs genes in rapeseed. Y2H assay was performed for protein interactions among 17 *Bna*CIPKs and 6 *Bna*CIPKs. Besides, the expression level of selected 12 *Bna*CIPKs and 6 *Bna*CBLs genes were analyzed under ABA, heat, cold, drought, and salinity stress via qRT-PCR. The findings revealed that CIPK and CBL families established a complicated signaling framework under several rapeseed abiotic factors.^27^

Another experiment was conducted to elucidate the function of MPK and MKK protein families in multiple stresses. The amino acid sequence was predicted for both MPK and MKK through sequencing and phylogenetic analysis. To unveil the cellular localization reporter gene, GFP was used. For protein–protein interactions among MPK and MKK families, the Y2H assay was run, and the results were further assessed through BiFC assay. Moreover, the expression of selected MPKs and MKKs under multiple stresses was evaluated by using RT-PCR. The result showed that 12 MPK and 7 MKK genes had been identified, which can be employed as a marker to generate stress-tolerance rapeseed.^173^ Genetic studies documented that ds-3 is responsible for dwarfism in rapeseed. The proteomic evaluation was performed for rapeseed to elucidate the role of genes, *BnaC07.RGA, BnaA06.RGA, BnaC09.RGA* and *BnaA09.RGA*. qRT-PCR and Y2H strategies were used for *BnaC09.RGA* and *BnaA09.RGA* gene expression and identified that both these genes have a considerable impact on the production of semi-dwarf rapeseed.^174^

### 2.4. Metabolomics: Are Metabolites Associated with the Closest Link to Phenotype?

To understand plant biochemistry at the organism and cellular level, metabolomics arising as a new era strategy to examine the whole metabolome of any crop plant. Due to its wide applications since the 1990s, metabolomics has been magnificently applied to plants to detect novel metabolites, genes and their metabolic pathways.^175^ Using metabolomics, GM crops can be evaluated in terms of their improved agronomic characters. Metabolomics consists of different signaling pathways, the interaction between proteins, the involvement of plant primary and secondary metabolites, and the epigenetic regulation process. Primary and secondary metabolites of plants are crucial to regulating biological and biochemical mechanisms.^175–177^

Metabolome analysis of high expression of the phospholipase C2 gene was studied in GE rapeseed and revealed that it has resistance against low temperature at a metabolomic level. GE plants show a remarkable increase in maltose and considerable enhancement in some free fatty acids, glycerol 3-phosphate and glycerol, stachyose, raffinose, and some flavonoids.^179^ For metabolome profiling gas chromatography-mass spectrometry (GC-MS) approach has been used for various rapeseed cultivars. Out of 162 compounds, metabolic profiling of 52 compounds has been successfully achieved. Different multivariate tools were applied, which showed remarkable variance among different rapeseed varieties.^176^ With the emergence of omics approaches, various researches have concentrated on elucidating the expression and function of several genes, proteins, and metabolites in rapeseed. Because of its commercial importance, oil-producing rapeseed has been subjected to MS tools to quantify and characterize rapeseed metabolome. Therefore, profiling of rapeseed metabolome aids in moving more deepen in plant biology and physiology to exploit an abiotic factor regulation.^179^ Recently,^140^ identified 41 differentially accumulated metabolites in the spring and 47 in the winter rapeseed ecotypes under cold stress. Notably, 81 metabolites primarily went to primary metabolites, including amino acids, organic acids, and sugars. They are suggesting that carbohydrates and amino acid compounds play a vital role in improving cold tolerance. Metabolomics is yet to be reported for several abiotic stresses in rapeseed.

#### 2.4.1. Metabolic QTL for Abiotic Stress Tolerance

**Figure 4.**
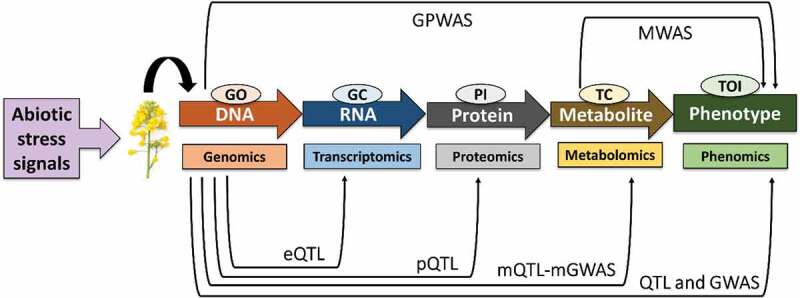
Systematic sketch of QTL mapping for gene expression together with molecular phenotype. The movement of particular knowledge is described from DNA-phenotype under abiotic stress signal. Notably, black arrows show that each molecular phenotype can be mapped onto the genome by using QTL mapping and GWAS techniques. However, MWAS does not demand particular knowledge to exploit the impacts of genetic deviant on metabolites. eQTL means epigenomic QTL, pQTL means proteomic QTL, mQTL means metabolomics QTL, mGWAS means metabolomic GWAS, MWAS means metabolome-wide association studies, GPWAS means genome-phenome wide association studies, GO means gene ontology, GC means gene co-expression, PI means protein interaction, TC means trait correlation, TOI means trait of interest. Modified from Razzaq et al. (2019).^176^

For phenotypical and morphological determination, various biomarkers have been developed. Thus, metabolic QTL (mQTL) and metabolic GWAS (mGWAS) approaches have been used to predict phenotype and establishment of metabotypes.^180^ For gene expression, protein and metabolome profiling are coupled with QTL mapping, as shown in Fig. 4. Genomic and metabolic markers are frequently correlated in mQTL mapping. In mQTL, a major difficulty arises in the detection of desired genes and metabolites. Therefore, a precise, measurable mapping of metabotypes is permitted to detect candidate gene.^176,179^ Metabolic levels of fatty acids, G3P, glycerol, and maltose significantly rose in GE plants in cold stresses by elevated concentrations of flavonoids, raffinose, and other sugars.^181^ Nuclear magnetic resonance (NMR)-based seed metabolites identification was carried out between two species, rapeseed and turnip. It was concluded that unsaturated fatty acids, sucrose, and sinapine are the most perspicacious metabolites influencing by changing the environment.^182^ The LC-TOFMS-based metabolomic and allopathic analysis was performed to understand the chemistry of rapeseed allelochemicals and their ability to hinder root and shoot growth.^183^

### 2.5. Phenomics: What Has Occurred?

The relationship between genotypes and phenotypes is very crucial for breeding programs. Plant phenomics is considered the phenotype of a plant or genotypic expression in a specific environmental condition.^184^ Additionally, a plant’s phenotype included several parameters that can be assessed through direct examination or by applying numerous analytical techniques and can also be defined by the association between environment and plant genotype.^185^ Phenotyping is still a massive task under abiotic stress conditions due to complex biosynthesis processes that govern several abiotic stress tolerance in plants.^185^ The significance of phenotyping has become apparent in the postgenomic era because the approaches used for crop improvement such as GWAS, GS, MAS, QTL mapping heavily depend upon the high-throughput phenotyping (HTP) in crops.^186^ Nonetheless, HTP technologies have been getting tremendous advancement. Data mining, interpretation, and storage strategies are automated, precise, accurate, and economical. Genetic dissection of various characteristics and detailed examination of plant structure and function permits studying the plant phenotypic expression.^188^

Recently, advanced HTP tools have been designed with multifunctional software coordination, consisting of X-ray tomography, hyperspectral imaging, and visible light imaging. This HTP allows the imaging of thousands of plants automatically at the same time. Many phenotyping centers have been set up in many countries (Table 5) and, notably, some unique QTLs have been documented in several plants.^187^ Recently, a few studies have been conducted using HTP in rapeseed. For instance, to speed-up the dissection of the dynamic genetic architecture of rapeseed growth and yield under different developmental stages.^189^ The phenotyping and genome-wide association mapping for the improvement of root architectural traits.^190^ Moreover, it has been summarized that the integration of holistic phenotyping tools can elucidate the functional gene polymorphism and explain the complex mechanisms responsible for abiotic stress tolerance in crop plants. Notably, in rapeseed, the utilization of different HTP tools under environmental stresses is still lacking.

**Table 5.** Globally available phenomics and phenotyping center to study the genotypic expression of plants under specific environmental conditions

Center name	URL	Country
PHENOPSIS- INRA	http://bioweb.supagro.inra.fr/phenopsis/	France
German Plant Phenotyping Network (DPPN)	https://www.ipk-gatersleben.de/en/phenotyping/	Germany
High-Resolution Plant Phenomics Center (HRPPC)	http://www.plantphenomics.org.au/HRPPC	Australia
National Plant Phenomics Center (NPPC)	https://www.plant-phenomics.ac.uk/	UK
European Plant Phenotyping Infrastructure (EMPHASIS)	https://emphasis.plant-phenotyping.eu/	Europe
Nordic Plant Phenotyping Network (NPPN)	https://nordicphenotyping.org/	Denmark
European Plant Phenotyping Network (EPPN)	https://eppn2020.plant-phenotyping.eu/EPPN2020	Europe
Phenome UK	https://www.phenomuk.net/	UK
PHEN-ITALY	http://www.phen-italy.it/index.php	Italy

## 3. An Overview of Modern Technologies for Developing Climate-resilient Rapeseed Plants

### 3.1. Transgenic Approaches

In the past few decades, transgenic methods have been widely used for developing abiotic stress tolerance plants. Genetic engineering for the development of stress resistance plants relies on the manipulation of genes responsible for stress regulation, which might be a way forward for enhancing plant health under stressful conditions. Assessment of the transgenic plants under stressful environments and regulation of physiological, biochemical, and cellular effects of the manipulated genes at the whole plant extent is still considered a persisting challenge to overwhelmed in rapeseed. So far, several transgenic rapeseed lines have been reported with improved abiotic stress tolerance (Table 6). For instance, transgenic rapeseed lines overexpressing *Arabidopsis CBF1/2/3* genes exhibited improved freezing tolerance due to the introduction of CBF-targeted orthologous rapeseed *Bn115* gene.^200^ Likewise, homologous overexpression of *BnCBF5* and *BnCBF17* enhanced freezing tolerance in transgenic lines.^153^ Whereas transgenic lines showed improved performance with *BnCBF17* as compared to *BnCBF5*, possibly due to higher expression of *cor* genes under cold stress.^153^ The transgenic lines expressing the *AtPLD-α-1* gene from *Arabidopsis thaliana* show it improves tolerance to drought and salinity stresses. It also reduced the H_2_O loss, improve biomass, and yield production under stress conditions.^192^ The hyperactive expression of the *Arabidopsis AtDWF4* gene elevated seed productivity and tolerance to heat, drought, dehydration stresses and enhances the seed yield in transgenic lines.^195^ The cloning of tobacco serine acetyltransferase (SAT; a rate-limiting enzyme for cysteine biosynthesis)-encoding gene (*NtSAT4*) generated high levels of glutathione and cysteine in transgenic rapeseed lines for improved heavy metals tolerance.^197^ In another study, the *Arabidopsis thaliana AtTrx-h2* gene improves the salinity tolerance by modulating antioxidant defense systems in transgenic lines.^198^ Several other examples are presented in Table 6.

**Table 6.** Stories of successfully developed transgenic rapeseed and transferred genes related to different abiotic stresses

Source plant	Gene	Coding protein or action mechanisms	Stress resistance	References
*Oryza sativa*	*OsNASI*	POD, dehydrogenase, GST, PSMB5 and RuBPco	Salinity	191
*Arabidopsis thaliana*	*AtPLD*-α*-1*	Phospholipase D-α-1	Reduced H_2_O loss, enhance biomass amassing and yield beneath drought and salinity conditions	191
N/A	*Cyp-11A1*	Bovine side-chain cleavage cytochrome P450scc	High temperature	194
Transgenic *Brassica napus*	*YHem1*	Promote the metabolism of endogenetic 5-ALA	Salinity	193
Transgenic *Brassica napus*	*BnPLC2*	Phosphatidylinositol-specific phospholipase C2 (PLC2]	Low temperature	178
Transgenic *Brassica napus*	*DREB*	DREB factor and improves the expression of several stress-related transcripts	Salinity and drought	196
*Arabidopsis thaliana*	*AtDWF4*	Brassinosteroid insensitive1 (BRI1)-EMS suppressor1 (BES1) and Brassinazole-resistant1 (BZR1)	Heat, drought, dehydration, enhance seed yield and also involved in biotic stress	195
*Arabidopsis thaliana*	*AtDFR*	dihydroflavonol-4-reductase	Improve salinity and drought tolerance by the accumulation of anthocyanins	197
*Nicotiana tabacum*	*NtSAT4*	Serine acetyl transferase (SAT)	Heavy metals	197
*Arabidopsis thaliana*	*AtTrx-h2*	h-type thioredoxins	Improves salt-tolerance and antioxidant systems	199
*Nicotiana tabacum*	*NtHSP17.6*	Heat shock proteins	Heat, drought, and salinity	201
*Brassica napus*	*BnKCS1-1, BnKCS1-2*, and *BnCER1-2*	–	Improve drought tolerance and cuticular wax	38

### 3.2. CRISPR/Cas System: The Promising Genome Editing Tool

**Figure 5.**
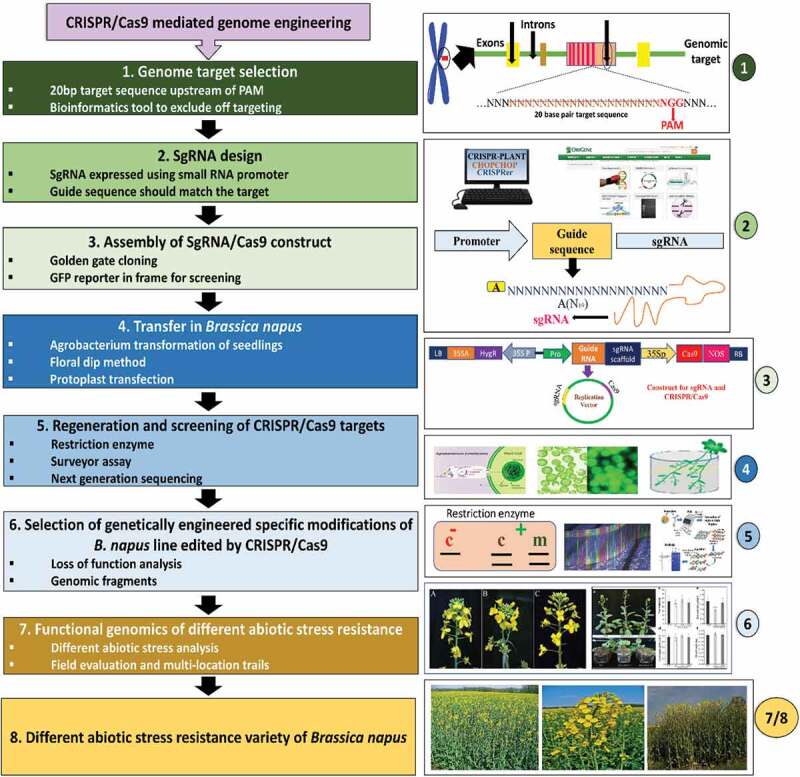
Step-by-step presentation of CRISPR/Cas9 mediated genome editing for the development of different abiotic stress resistance varieties of rapeseed. Each step (1–8) shows some basic sub-steps within the box (left side). In contrast, the right side indicates the practically on-table illustrations of the steps involved in the CRISPR/Cas9 mediated genome editing in rapeseed

Genome editing is an efficient tool for crop improvement either by the loss-of-function, the gain-of-function, or a multiplex genome editing methods. Thus, clustered regularly interspaced short palindromic repeats**/-**associated proteins (CRISPR/Cas9) have come out as a robust GE strategy and are considered a fast, modest, and multipurpose procedure for GE and development of a transgene-free end-product.^48,202^ The step-wise presentation of CRISPR/Cas9 based GE for the evolution of different stress-tolerance varieties of rapeseed is described in Fig. 5. Nevertheless, CRISPR/Cas9 mediated GE’s mutation efficiency was observed and examined the heritability and pattern of gene manipulations in rapeseed. Interestingly, no off-target mutation was produced in mutated lines, and this investigation has opened new horizons for biotechnological applications in rapeseed for the development of stress-tolerance cultivars.^48^ The difficulty of the rapeseed genome and gene redundancy is the main limiting factor for simultaneous mutagenesis of several homologs in the first progeny. Furthermore, the widespread identification of mutant plants is time-consuming through conventional approaches.^203^ Recently, an expression cassette for sgRNA targeted five homologous genes, such as *BnSPL3-Cnn, BnSPL3-C4, BnSPL3-C3, BnSPL3-A4*, and *BnSPL3-A5* have been developed. High-throughput sequencing investigation disclosed a very high knock out the efficiency of about 96.8% to 100% by CRISPR/Cas9.^203^

CRISPR/Cas9 based GE has excellent power in breeding programs to develop improved varieties for agronomic traits in many crops. However, the early exploitation of this tool in enlightening agronomic traits of rapeseed, e.g., the *FAD2* gene, which controls the enzyme that catalyzes oleic acid desaturation in rapeseed,^204^ the *BnLLA10* gene which controls the lobed-leaf shape and regulates salt tolerance in rapeseed.^205^ Moreover, the knockout of two *BnaMAX1* homologs advances plant architecture and upsurges yield,^206^ precise editing of *CLAVATA* genes normalizes silique development,^207^ pod shatter resistance by the multiplex editing of INDEHISCENT homologues,^208^ and JAGGED genes,^209^ flowering time and plant architecture by knockout of *BnaTFL1* gene.^210^ In a recent study, the gain-of-function mutant (*bnaa6.rga-D*) showed boosted drought tolerance, and its stomata were oversensitive to ABA signaling, whereas *bnarga* (loss-of-function mutant) owned compact drought tolerance and less sensitivity to ABA signaling.^211, 212^ Notably, in rapeseed, the CRISPR/Cas9 system’s utilization for the development of abiotic stress-tolerant rapeseed is still lacking and yet to be documented under several stress conditions.

## 4. Persisting Bottlenecks in the Production of Climate-resilient Rapeseed

**Figure 6.**
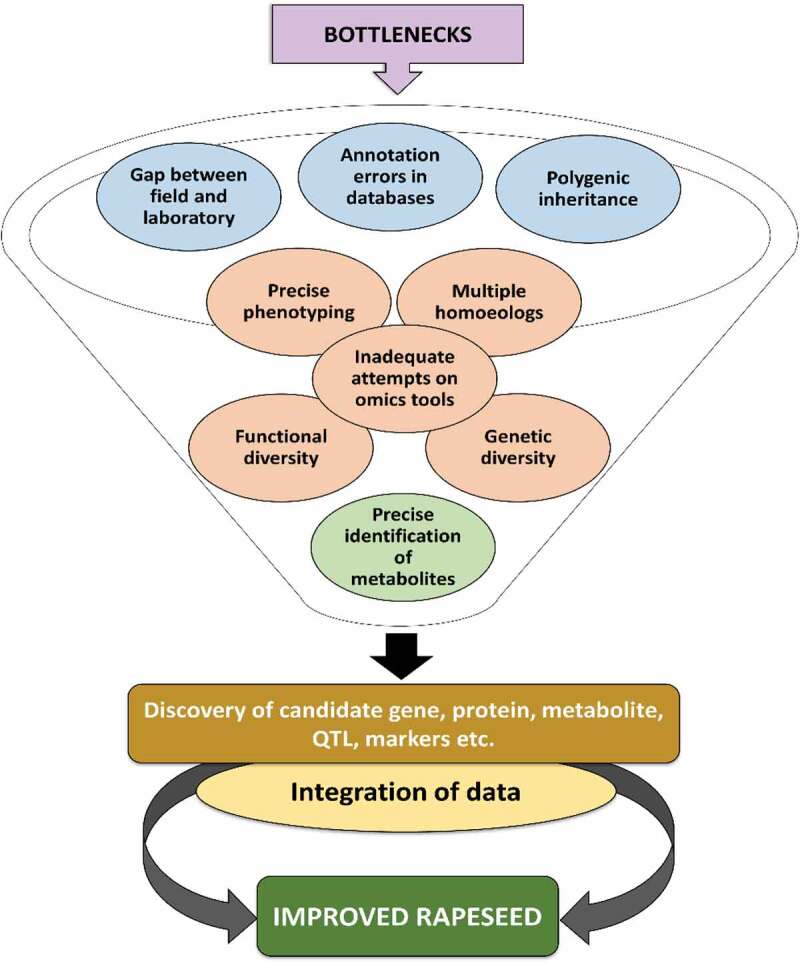
Persisting bottlenecks in the utilization of omics approaches to develop climate-resilient rapeseed. The dismissal of these bottlenecks using different molecular tools will help us to exploit the new manifesto to develop abiotic stress tolerance rapeseed; and successively will assure global food security

Although marvelous advancement has been achieved in the biotechnological era, many questions and bottlenecks presently restrict the implementation of omics approaches for abiotic stress tolerance studies in rapeseed. Therefore, few bottlenecks demand to be abundantly handled to decipher the potential of omics tools for rapeseed (Fig. 6). Based on the available literature, there are very few abiotic stress tolerance rapeseed genotypes developed via omics tools. However, after the rapeseed genome sequence, researchers pay attention to identify candidate genes and regulators to improve abiotic stress tolerance in rapeseed. Still, few genes have been reported for abiotic stresses.

Moreover, polygenic inheritance in abiotic stress tolerance rapeseed is still acting as a major bottleneck in conventional breeding programs. The misplacement of genetic divergence during the domestication of rapeseed is still considered a significant bottleneck among the existing ones. There is still a noticeable gap between the field and laboratory. However, less genetic diversity directly leads to less functional diversity. The biggest question is to utilize wild diversity more quickly, which can be utilized to explore genomic parts of loci related to essential QTL for abiotic stress tolerance in rapeseed.

Even though it is viable to identify thousands of metabolites, however, the precise identification of leftovers as the utmost bottleneck in plant metabolomics investigations. Insufficient knowledge and inadequate attempts on omics tools, mainly metabolomics, proteomics, and annotation mistakes in databases, are a significant barrier for functional characterization, and still act as significant bottlenecks in genome analysis.

However, the integration of newly emerged molecular platforms for identifying complex QTLs has remained a key restriction due to restricted potential to phenotype the crops precisely. Together, precise phenotyping concealed by native habitat is still a big phenotyping-bottleneck in several diverse development stages, including translation of genotype to phenotype. Another significant bottleneck is multiple homoeologs of a single gene in the rapeseed genome; in this regard, more homoeologs need to be targeted using GE tools. Thus, the dismissal of these bottlenecks using molecular tools will help us to exploit the new manifesto to develop stress resistance rapeseed; and successively will assure global food security.

## 5. Concluding Remarks

Rapidly changing climate is the main reason for several environmental stresses in the current era. Among them, abiotic stresses immensely affect rapeseed growth and production globally. These stresses may cause irregularities and a decrease in crop yield parameters. They can execute physiological, morphological, and molecular variations that deleteriously disturb production, growth, and final rapeseed yield. Therefore, it is vital to explore the stress-responsive mechanisms to enhance rapeseed production and quality under harsh environmental conditions. To solve this persisting issue, omics tools have demonstrated the most outstanding biotechnological applications to develop abiotic stress-tolerance crops.

Omics, GE tools, and molecular breeding strategies have been extensively used to elucidate the complex biological mechanism and pathways regulating abiotic stress-tolerance in rapeseed. The multi-dimensional omics studies have gathered numerous datasets at the transcriptome, proteome, and metabolome level to unveil various molecular, physiological, and metabolic pathways related to abiotic stress-tolerance in rapeseed, which open ups new horizons for future investigations. However, few bottlenecks demand to be abundantly handled to decipher the potential of omics tools for rapeseed and other crops (Fig. 6). However, after the rapeseed genome sequence, researchers pay attention to identify candidate genes and regulators to improve stress tolerance in rapeseed. Still, few genes have been reported for multiple abiotic stresses. More work needs to be carried out to fully explore the beneficial role of omics in abiotic stress tolerance.

Molecular markers are the most powerful technology in plant molecular biology to determine the genetic variations in rapeseed at the polyploidy level. Additionally, with the accessibility of the rapeseed genome sequence, we can detect candidate genes related to several abiotic stresses in modern breeding programs. High-throughput sequencing technologies and EST construction for rapeseed are very promising for identifying SNPs for various abiotic stress-related QTLs. The applications of SNPs in rapeseed are very diverse due to its complex genome. Continuous improvements in NGS and genotyping techniques permit genotyping by sequencing, and SNPs will offer valuable information for rapeseed scientists. Moreover, genomic selection tools, along with HTP stages for selecting huge populations, permit more insights.

## 6. Future Directions

Genome editing via the CRISPR/Cas system and engineering stress-associated genes should be considered one of the most promising research directions to boost stress tolerance. Likewise, the engineering of metabolic pathways can open novel gaps for climate-resilient development, ready to grow rapeseed plants. Speed breeding has developed as a time-saving tool to expand plant growth and development under preferred conditions with enhanced stress tolerance. In the future, speed breeding will boost the genetic understanding and allow abiotic stress-tolerance rapeseed as established in other crops. Nevertheless, the CRISPR/Cas9 system can be integrated with speed breeding to modernize world rapeseed production. Shortly, the combination of omics, genome editing, and speed breeding can speed up the rapeseed production with improved traits and increased abiotic stress tolerance. On the other hand, another emerging approach, “synthetic biology” can be mainly applied to plant biology and engineering methods to develop climate-smart rapeseed plants.

## Supplementary Material

Supplemental Material
