# Supplementary material for: Omics: The way forward to enhance abiotic stress tolerance in Brassica napus L
Source: GM Crops Food. 2021 Jan 19;12(1):251–81. doi: 10.1080/21645698.2020.1859898 (PMC7833762; doi:10.1080/21645698.2020.1859898)
Supplement: Supplemental Material [file KGMC_A_1859898_SM8871.docx]

**Supplementary Table 1.** Till date, progress of completed *B. napus* genome sequencing and resequencing project. Modified from Snowdon et al. (2012).

| **Genotype** | **Type** | **Purpose of sequencing** | **Sequencing method** | **Consortium/Country** | **Researchers contact** |
| --- | --- | --- | --- | --- | --- |
| DH12075 | Spring canola | *B. napus* reference assembly | Illumina-454 | CAN-SEQ | Isobel Parkin. Agriculture and Agri‐Food Canada |
| Darmor DH | Winter oilseed rape | *B. napus* reference assembly | Illumina-454, Sanger | France | Boulos Chaloub. INRA, Évry, France |
| Zhongshuang 11 | Spring oilseed rape | *B. napus* reference assembly | Illumina-454 | China | Shengyi Liu, OCRI-CAAS, Wuhan, China |
| Tapidor DH | Winter oilseed rape | *B. napus* reference assembly | Illumina-454 | China and UK | Jinling Meng, Huazhong Agriculture University, Wuhan, China |
| Ningyou 7 | Chinese oilseed rape | *B. napus* reference assembly | Illumina-454 | China and UK | Jinling Meng, Huazhong Agriculture University, Wuhan, China |
| 51 lines | Diverse *B. napus*-association mapping | Whole-genome resequencing | Illumina | Germany | Rod Snowdon, Justus Liebig University, Giessen, Germany |
| 123 lines | Diverse *B. napus* | Leaf transcriptomes | Illumina | UK | Ian Bancroft, John Innes Centre, Norwich, UK |
| 517 | Species‐wide *B. napus* diversity collection | Restriction‐associated DNA (RAD) | Illumina | ASSYST/Germany | Benjamin Stich, Max Planck Institute for Breeding Research, Cologne, Germany |
| Express 617, V8 plus 94 ExV8‐DH lines | Winter oilseed rape mapping population | RAD | Illumina | Germany and China | Rod Snowdon, Justus Liebig University, Giessen, Germany |
| 500 lines | Diverse winter‐type *B. napus* | Sequence capture of 40+ flowering regulatory genes | Illumina | Germany | Rod Snowdon, Justus Liebig University, Giessen, Germany |
| 10 inbred lines | 6 winter and 4 spring | Sequence capture of meta‐quantitative trait loci regions | Illumina-454 | Chile and Canada | Federico Iniguez‐Luy, Agri aquaculture Nutritional Genomic Center (CGNA), Chile |
| Darmor-bzh | European winter oilseed | The consequences of its recent duplication | 454 GS-FLX+ Titanium and Sanger sequence | France | Boulos Chaloub. INRA, Évry, France |
| Eight oilseed rape lines, including four SWORs (ZS11, Gangan, Zheyou7 and Shengli), two WORs (Tapidor and Quinta) and two SORs (Westar and No2127) | Spring and winter | Pan-genome | 454 GS-FLX + Titanium and Sanger sequence, NGS or medium-coverage PacBio single-molecule real-time (SMRT) sequencing | China | Liang Guo, Huazhong Agriculture University, Wuhan, China |
